# Inorganic Chemistry

**Published:** 1893-04-01

**Authors:** 


### INORGANIC CHEMISTRY.

For the solitary student the small volume on "Inorganic Chemistry,"† published in the "Catechism Series" will be found extremely useful. Of course, in the limited space of sixty pages the subject cannot be fully treated, but the questions and answers seem, on the whole, to be of the kind that would probably be asked at an examination, and hence this volume will be found useful to test a candidate's knowledge just before he presents himself to an examining board. We regret, however, to find new names introduced for some old friends. Thus, why should "Arsine," "Phosphine," and "Stibine" be substituted for the familiar arsiniuretted, phosphuretted, antimoniuretted hydrogen respectively? The new names are no doubt shorter, but the old ones possess the great merit of being descriptive. We also note that the formula for Feric Chloride is incorrectly given as $Fe\,cl_3$ instead of $Fe_2\,cl_6$ and "Aqua Regia" is given as an alternative name for Nitric Acid alone. Moreover, as there are two oxides of Arsenic, it can only be misleading to the student to call Arsenic Pentoxide, Arsenic Oxide. It is a pity that such useless changes have been introduced into an otherwise excellent little book.

*"Aids to Gynæcology," p. 116. A. S. Gubb, M.D., Paris. (Baillière, Tindall, and Co., London.)

† "Chemistry, Part I.—Inorganic." Catechism Series. (E. and S. Livingstone, Edinburgh. Price 1s.)

